# DC-electrical penetration graph waveforms for *Dalbulus maidis* (Hemiptera: Cicadellidae) and the effects of entomopathogenic fungi on its probing behavior

**DOI:** 10.1038/s41598-023-48779-x

**Published:** 2023-12-12

**Authors:** Nathalie Maluta, Thiago Castro, João Roberto Spotti Lopes

**Affiliations:** 1 Department of Entomology and Acarology, Luiz de Queiroz College of Agriculture, University of São Paulo, C.P. 9, Piracicaba, SP 13418-900 Brazil; 2 Koppert Biological Systems, Rodovia Margarida da Graça Martins s/n - Km 17.5, Piracicaba, SP 13400-970 Brazil

**Keywords:** Entomology, Fungal host response

## Abstract

The corn leafhopper *Dalbulus maidis* is an important transmitter of viruses and bacteria to maize plants. Data on the stylet probing and ingestion behavior of *D. maidis*, gathered using the DC-electrical penetration graph (DC-EPG) technique, are limited. The increasing prevalence of this pest and the diseases that it transmits in maize crops heighten the importance of studying how control tools affect the probing behavior of these insects, to reduce or prevent the chances of pathogen transmission and the damage from feeding. Our study recorded stylet activities of *D. maidis*, using a DC-EPG system and compared the appearances of waveforms with those from published AC and AC-DC EPG studies. All types of systems produced similar waveform appearances; therefore, we used the waveform nomenclature previously published. We also determined the effects of the entomopathogenic fungus *Cordyceps javanica* on the probing behavior of *D. maidis* at different time points after the fungus was applied by spraying. Forty-eight hours after the insects were sprayed, the effects were pronounced, with significant disruption of the stylet activities in phloem and non-phloem phases. Our study indicated that this commercial microbiological product, with the active ingredient *C. javanica*, can alter the probing behavior of *D. maidis* and may be helpful in management of the vector.

## Introduction

The corn leafhopper, *Dalbulus maidis* (DeLong & Wolcott) (Hemiptera: Cicadellidae), is an important pest species of maize (corn) plants (*Zea mays* L.). This leafhopper is widely distributed in the Americas, from the southern United States to northern Argentina. In Brazil, *D. maidis* uses only maize plants as a host, and little is known about its survival mechanisms in the absence of these plants.

*Dalbulus maidis* is important not only because of the direct damage it causes from phloem sap sucking, but mainly the indirect damage from transmission of phytopathogens. These pathogens include bacteria limited to the phloem vessels, such as the etiological agents of ‘Maize bushy stunt (MBS) phytoplasma’ (MBSP) and ‘Corn stunt spiroplasma’ (*Spiroplasma kunkelli* Whitcomb)^1,2^. This species of leafhopper also transmits the maize stripe virus, maize rayado fino virus (RFV), which is also limited to phloem vessels^3^.

In consideration of their importance for maize crops, controlling these leafhoppers is necessary to reduce populations and, mainly, transmission of phytopathogens to new host plants. Chemical insecticides are by far the most often used, with several active ingredients registered for control of this species. Only two entomopathogenic fungi, *Beauveria bassiana* (Bals.-Criv.) Vuill. and *Cordyceps javanica* (Wize) (formerly *Isaria fumosorosea* and *Cordyceps fumosorosea*)^4,5^ are registered. The latter is a generalist species, commercially available and with high control potential for sap-sucking insects^6–9^.

The stylet probing behavior of sap-sucking insects can be monitored using the electrical penetration graph (EPG) technique, allowing the activities of the stylets in different plant tissues to be viewed on a monitor in real time^10,11^. This technique generates waveforms with different characteristics such as, voltage level, frequency, and amplitude. The waveforms can be correlated with biological activities, including insecticide effects on the stylet actions, and transmission of pathogens, among others^8,9,12,13^.

In the present study, we evaluated the stylet activities of *D. maidis* after application of the entomopathogenic fungus *C. javanica* using the Direct Current (DC)-EPG system. We then compared waveform appearances we observed with previous, published information on the characterization and correlations of the waveforms studied by Carpane et al.^14^ (AC-DC EPG system) and Carpane & Catalano^15^ (DC EPG system). We hypothesized that *C. javanica* would cause physiological and behavioral changes in *D. maidis* due to the fungal infection process. We tested whether the fungus could alter the stylet activities of *D. maidis* at different time points after spraying. The results would indicate whether this biological-control agent could be helpful in reducing the feeding of corn leafhoppers and transmission of phytopathogens in maize crops.

## Material and Methods

### Biological material: corn leafhopper *Dalbulus maidis* colony and maize plants

A colony of healthy *Dalbulus maidis* was initially established from insects collected on maize plants in the municipality of Jardinópolis, state of São Paulo (SP), Brazil (20.912931S; 47.896399W) and maintained in screened cages with an aluminum frame (35 × 35 × 53 cm) and acrylic door, containing maize plants (commercial Hybrid LP2020-LongPing High-Tech Seeds and Biotechnology, Jardinópolis) in a climate-controlled greenhouse (25 ± 2 °C; 60–70% RH) under natural light at ESALQ-USP, Piracicaba, SP. For the waveform correlation and the probing behavior study we used healthy maize plants on vegetative stage (V3-V4: 3–4 fully expanded leaves; ~ 25 days old), and each insect and plant was used only once in the EPG assays. All plants used in this study are maize plants from commercial seeds (Hybrid LP2020-LongPing High-Tech Seeds and Biotechnology, Jardinópolis), and as they are a cultivated species there is no need to deposit a specimen in an herbarium.

### EPG waveforms comparison

For the experiment to compare appearances of waveforms generated by *D. maidis* stylet activities using DC-EPG, healthy females (7–15 days old), from the colony maintained on maize plants, were enclosed in a glass tube placed in an ice bath for 4–6 min to facilitate manipulation, and then immobilized using a vacuum chamber under a dissecting microscope (Carl Zeiss Suzhou Co., Ltd; Germany). Then, a gold wire (3 cm in length, 18 µm in diameter; EPG Systems, Wageningen, The Netherlands) was attached to the leafhopper pronotum with a droplet of water-based silver glue. The opposite end of the gold wire was glued to a thin copper wire (2 cm in length), which was connected to the EPG head state amplifier. Another copper electrode (10 cm long, 2 mm wide) was inserted into the soil of the plant.

After a 1 h starvation period, each corn leafhopper was placed individually on the abaxial surface of a maize leaf. The EPG waveforms were recorded for 10 h inside a Faraday cage (for electrical noise isolation) in a climate-controlled room (25 ± 1 °C), using a Direct Current eight-channel EPG device, model Giga-8d, with Stylet + software for Windows (EPG Systems, The Netherlands). In addition to associating DC-EPG waveforms for this species, means were calculated for the number of waveforms (NWEI: number of waveform events per insect), the total duration of each waveform (WDI: summed waveform duration (min) per insect)^16,17^. Parentheses in the WDI variable name above indicate information about calculating the variable not included in the variable name. The following sequential variables were tested: “Total duration of phloem phase”; “Time to 1st probe from start of EPG”; “Number of probes to the 1st phloem”; “Time from start of EPG to 1st phloem”; “Number of probes after 1st phloem”; “Time from 1st probe to 1st phloem”. Twenty-two records from different insects were analyzed.

The waveforms generated by leafhopper feeding behavior on maize plants were compared with waveforms previously described for *D. maidis* by Carpane et al.^14^, using the EPG AC-DC system, and by Carpane & Catalano using the DC-EPG system^15^, studied the host plant resistance of maize hybrids, compared the waveforms obtained with the study of Carpane et al.^14^. The recorded was adjusted on gain 50x, and the voltage was adjusted individually for each EPG channel, keeping the output voltage around 2–3 V on the monitor screen.

In addition, to these authors, other studies with species of the cicadellid subfamily Deltocephalinae were used to support our comparison of waveforms from feeding behavior with biological activities^18–22^. These studies used all types of EPG systems (AC, DC, and AC-DC). Comparison was performed based on voltage, frequency, amplitude, and shape of the waveforms. The nomenclature of the waveforms follows that of Carpane & Catalano^15^.

### Stylet activities of *Dalbulus maidis* sprayed with biological product

The experiments related to the stylet probing behavior of *D. maidis* were carried out after application of the commercial microbiological product. Before the probing-behavior assays, adult *D. maidis* (7–15 days after emergence) from the maintenance colony were placed in a glass tube in crushed ice for 3–5 min to immobilize them. In groups of 30 insects (not sexed), they were positioned in the center of a Petri dish covered with filter paper and sprayed with a solution of the biological insecticide Octane® (*Cordyceps javanica*, strain ESALQ 1296, Koppert Biological Systems Brazil) at the dose recommended by the manufacturers (320 µl of commercial product/100 ml of water). Approximately 1.5 ml of solution was sprayed on each group of leafhoppers (spraying was performed at 1 m between the spray bottle and the insect).

The time points used after spraying were: 0, 15, 30, 48, 72, 96, and 120 h, according to the general biological phases of the fungus (adhesion, germination, penetration, vegetative growth)^23,24^. Each time point was composed of a treatment with *C. javanica* and its control with water, and the tests were performed separately, in a climate-controlled room (25 ± 1 °C).

Insects were prepared for the EPG assay as described by Maluta et al.^9^. Then, each leafhopper was individually positioned on the abaxial face of a fully expanded maize leaf (Hybrid LP2020). Insect probing behavior was monitored for 10 h inside a Faraday cage, in a climate-controlled room (25 ± 1 °C), using the same DC-EPG device as above. Twenty replicates (one insect was considered one replicate) were analyzed per treatment at each time point.

To ensure fungal infection, after the EPG recordings, the insects were placed in a humid chamber for sporulation, and only leafhoppers with confirmed mortality caused by *C. javanica* were used in the analyses. As a control, the same procedure was used, except that the leafhoppers were sprayed only with water.

### Statistical analysis

EPG waveforms observed were similar to waveforms described by Carpane et al.^14^ and Carpane & Catalano^15^, as detailed below in “Results”. Therefore, the same waveform naming convention as established in those papers was used herein.

Insect behavior for fungal treatments (treated and untreated leafhoppers) were compared within the same time point. A combined analysis was not performed because the insects were not the same age at the different time points. For analysis, only the records for which: (a) the insects performed probing activity in at least 5 of the 10 h of recording; (b) leafhoppers were glued to the wire and on the plant at the end of the recording; (c) insects with confirmed infection by *C. javanica* or negative for the control treatment.

Before the analysis, normality and homogeneity of variance were confirmed, and when necessary, the data were transformed with ln (x + 1) or √(x + 1) to reduce heteroscedasticity and improve normal distribution. The EPG variables were analyzed with a parametric Student’s *t* test (data with Gaussian distribution) or a nonparametric Mann–Whitney *U* test (data with non-Gaussian distribution). All data were analyzed using IBM Statistics SPSS 22.0 software^25^.

### Ethics approval and consent to participate

The experimental research and collection of plant and insect materials of this study comply with the relevant institutional, national, and international guidelines and legislation.

## Results

### DC-EPG waveforms and biological activities of *Dalbulus maidis*

**Figure 1:**
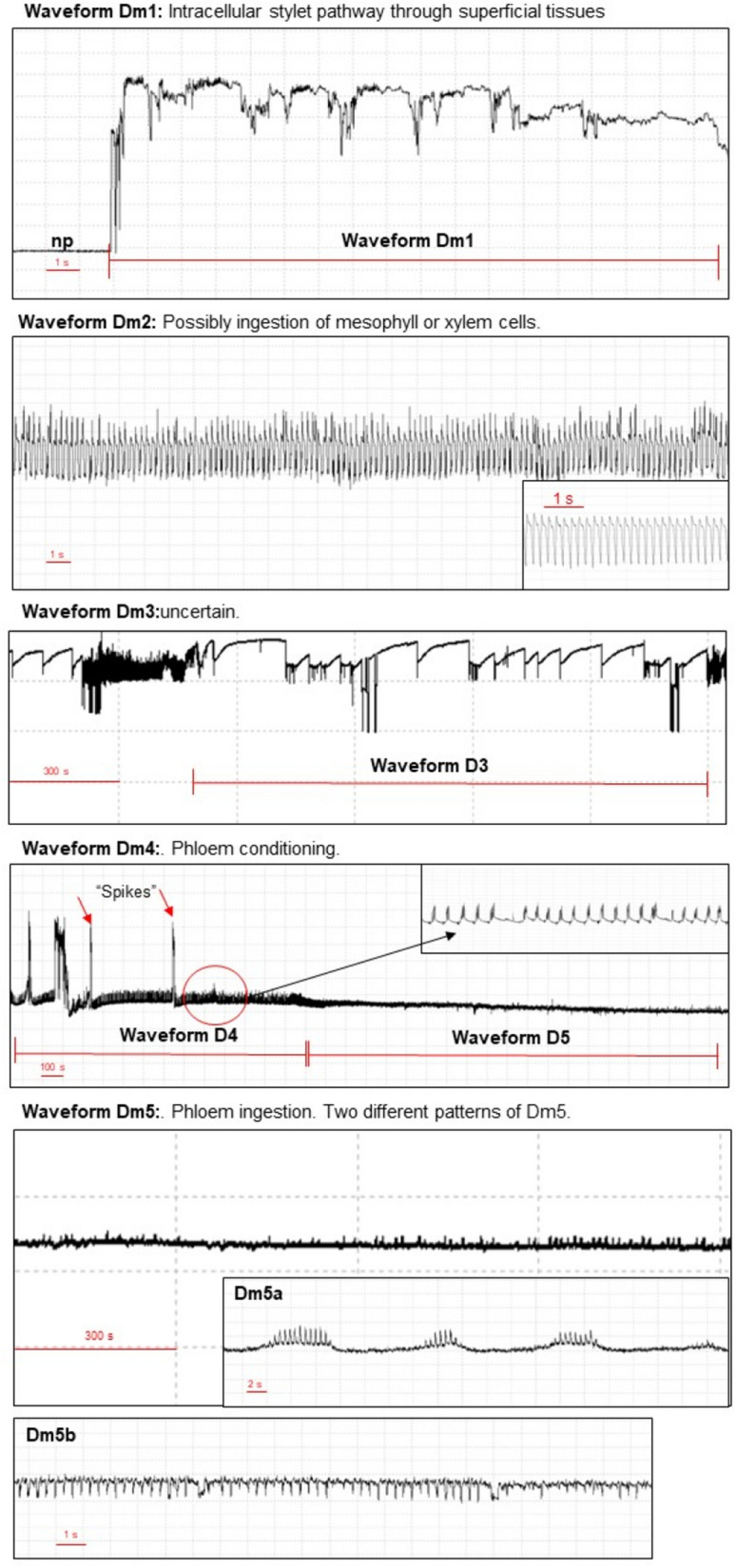
EPG-DC waveforms observed during probing of *Dalbulus maidi*on maize plants. Overview of five probing behaviors, DM1-D5.

When females of *D. maidis* probed on maize plants, five different waveform types were observed (Table 1; Fig. 1). The waveforms identified were:

**Table 1:** Mean (± ESM) of non-sequential and sequential EPG variables for 10-h recordings of the probing behavior of *Dalbulus maidis* on maize plants (*Zea mays*).

EPG variables	NWEI^a^ (n = 22)	WDI (min)^b^ (n = 22)
Non-sequential EPG variables		
Probing	22.23 ± 3.92	523.75 ± 8.07
Np	22.41 ± 3.91	76.25 ± 8.07
Dm1	39.91 ± 5.77	93.90 ± 10.93
Dm2	22.36 ± 2.25	172.97 ± 21.41
Dm3	11.86 ± 2.07	114.06 ± 21.62
Dm4	3.45 ± 0.97	39.04 ± 12.0
Dm5	1.32 ± 0.30	103.77 ± 24.96
Sequential EPG variables		
Number of probes to the 1st Dm4		9.77 ± 1.71
Number of probes after 1st phloem		12.45 ± 3.95
Time to 1st probe from start of EPG (min)		5.53 ± 1.62
Time from start of EPG to 1st phloem (min)		313.97 ± 46.76
Time from 1st probe to 1st phloem (min)		308.44 ± 46.49

^a^(NWEI): Mean number of waveform event per insect, ^b^(WDI): (Total or summed) waveform duration per insect (minutes).

#### Probing

**Figure 2:**
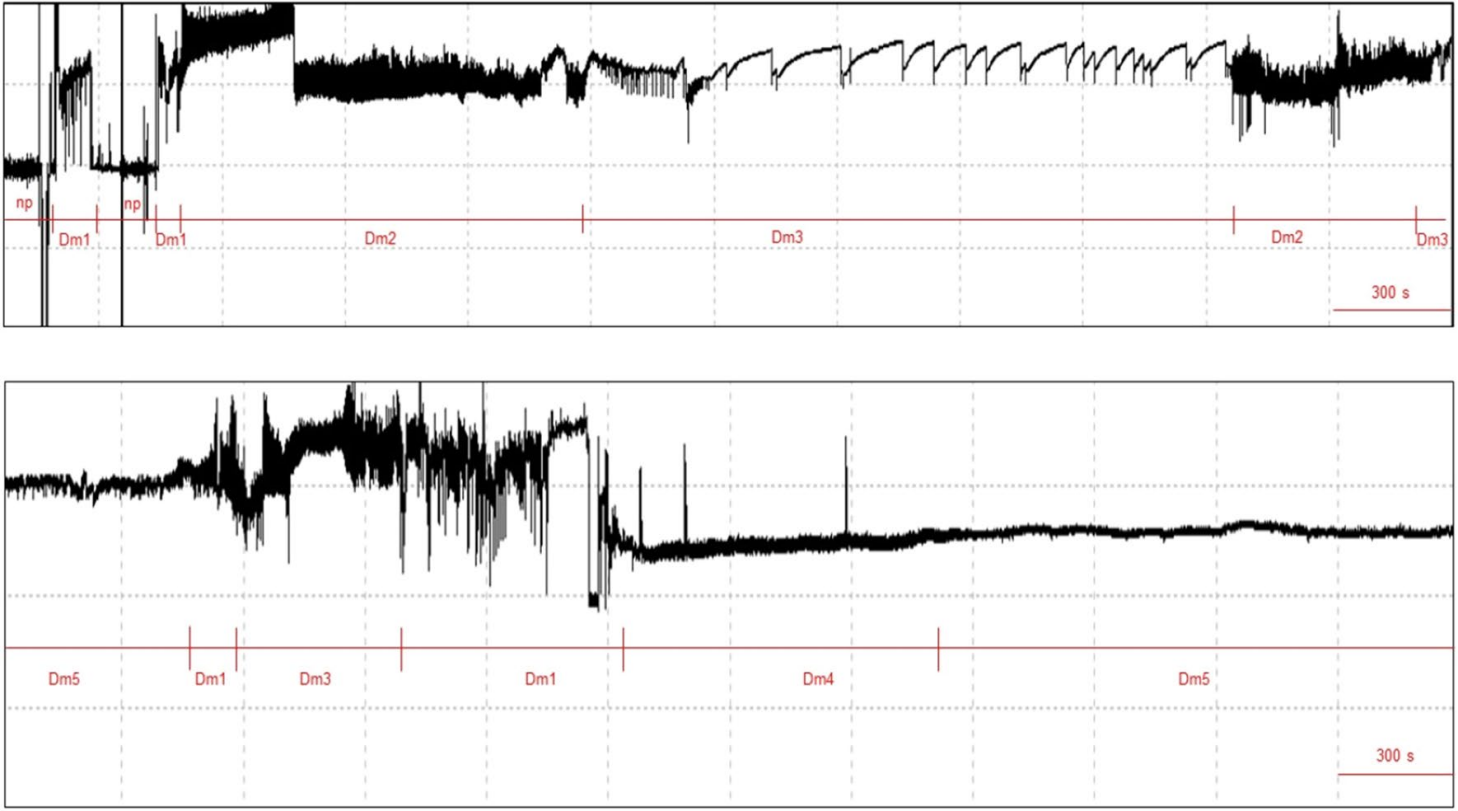
Samples of major waveforms displayed during 1-h probing of *D. maidis* females stylet probing on maize plants.

The probing behavior does not refer to a specific behavior but is the sum of all stylet activities within plant tissues performed by insects (Fig. 2).

#### Np

Non-probing, when the stylets were outside the plant tissue, so the insects were not probing.

#### Waveform Dm1

This is the first waveform that occurs after np, with a sudden increase of voltage occurring with variable frequency and positive voltage. It is associated with the pre-phloem phase, during intercellular penetration of the stylets through superficial tissues (epidermis, mesophyll, and parenchyma cells).

#### Waveform Dm2

Likely ingestion from non-phloem tissue such as mesophyll cell or xylem element. The frequency varies between 4 and 9 Hz, the duration is variable, and the voltage level is positive. There is secretion of honeydew by the corn leafhopper during this waveform, observed during the recordings, and the waveform shape is similar to waveform G (xylem) in aphids and whiteflies.

#### Waveform Dm3

The frequency varies between 0–1 Hz; the duration is variable, and the voltage level is positive. The waveform Dm3 is characterized by sequences of sharp peaks with negative deflections of high amplitude, and always occurs after Dm1 or Dm2. As observed by other investigators, this waveform is correlated with unidentified biological activity. According to the hypothesis of Carpane & Catalano^15^, this waveform might be correlated with 'stylet work'.

#### Waveform Dm4

**Figure 3:**
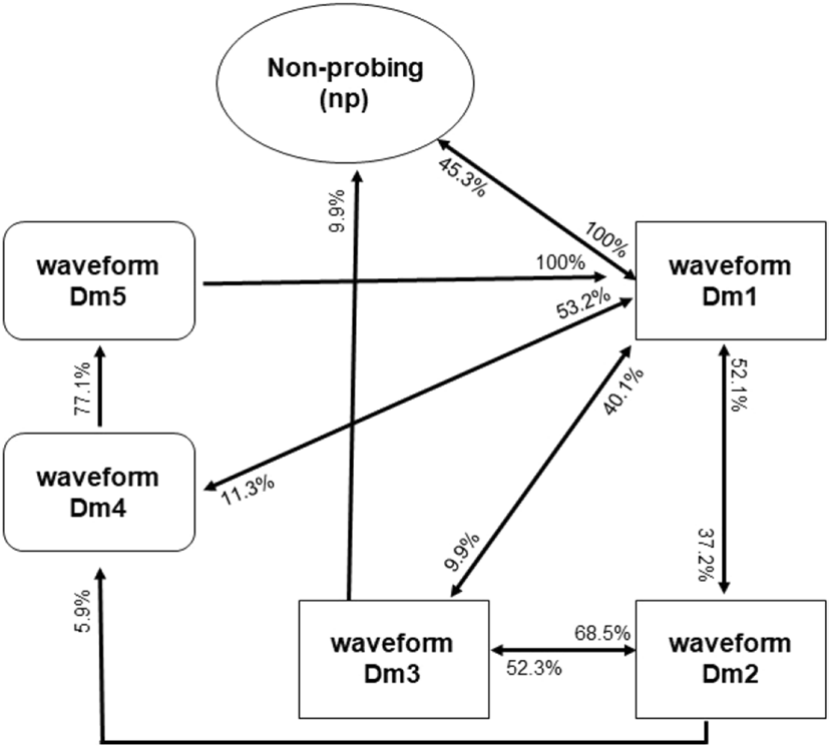
Transition scheme (kinetogram) of waveforms events for *Dalbulus maidis* on maize plants during the 10 h EPG recording. The percentage near the arrows correspond to the likelihood of a certain waveform being followed by another waveform type.

Phloem conditioning, wherein salivation into the phloem and egestion occur, correlated with inoculation of *Spitoplasma kunkelii*. The frequency of this waveform varies between 1 and 3 Hz, and the voltage level is negative. The waveform shape is similar to waveform E1 in aphids, whiteflies, and psyllids^10,11,26–28^. Waveform Dm4 always occurs after Dm1, Dm2 or Dm3, and in general is followed by Dm5 (Figs. 1, 2 and 3).

#### Waveform Dm5

Waveform Dm5 always occurs after Dm4. The frequency of this waveform varies between 1 and 3 Hz, and the voltage level is negative. Two distinct patterns can occur within Dm5, one after the other in sequence, called Dm5a and Dm5b; repeated sequences of Dm5a and Dm5b separated by Dm4 may occur (Figs. 1, 2 and 3).

Waveforms Dm1, Dm2, and Dm3 are associated with activities performed outside the phloem tissue, or pre-phloem activities. Waveforms Dm4 and Dm5 are associated with stylet activities in the phloem tissue.

Over nearly half of the total recording time (600 min), the insects performed activities associated with waveforms Dm2, Dm3, and Dm5. As observed for other insects, the number (NWEI) of times that insects perform waveforms associated with the phloem (Dm4 and Dm5) is small compared to other waveforms. However, they perform these activities for long durations (Table 2).

**Table 2:** Waveform characteristics and biological meaning of *Dalbulus maidis* on DC-EPG system on maize plants.

Waveform	Characteristics	Waveform transition	Activity
Np	-Baseline -Stylets out of plant tissue	Before waveform Dm1	-Non-probing
Dm1	-Positive voltage -Varies in frequency -Similar to aphid/whitefly waveform “C”, with variable frequency -Sudden increase in voltage -High amplitude	The first waveform, after np	-Stylet pathway
Dm2	-Positive voltage -Varied in duration -Frequency 5–9 Hz -High amplitude -The waveform shape is like waveform G (xylem) for aphids and whiteflies	After Dm1 or Dm3	-Possibly active ingestion of mesophyll or xylem cells -There is secretion of honeydew by the corn leafhopper, during this waveform
Dm3	-Positive voltage -Frequency 0–1 Hz -Characterized by sequences of sharp peaks with negative deflections of high amplitude	After Dm1 or Dm2	Unknow
Dm4	-Negative voltage -Frequency 1–3 Hz -Low amplitude	After Dm1, Dm2 and always before Dm5	-Phloem conditioning
Dm5	-Negative voltage -Frequency 1–3 Hz -Low amplitude	Always after D4	-Probably phloem ingestion; 2 distinct patterns (Dm5a and Dm5b) -There is secretion of honeydew by the corn leafhopper, during this waveform

### Effect of microbiological insecticide on the stylet probing behavior of *Dalbulus maidis*

#### 0 h after spraying

**Figure 4:**
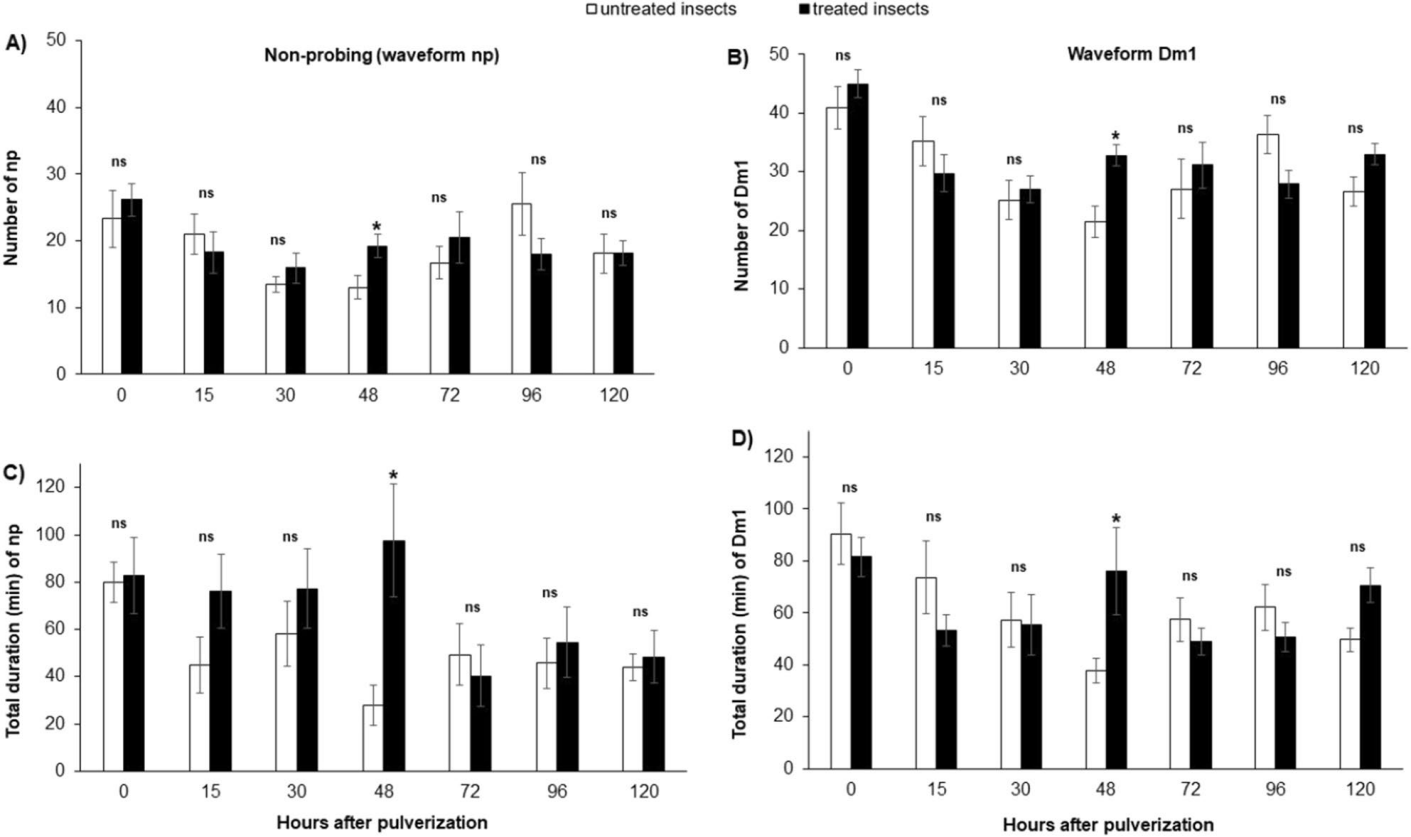
Mean number of waveform event per insect (NWEI) and total waveform duration per insect (WDI) of non-probing (**A**,**C**) and waveform Dm1 (stylet pathway) (**C**,**D**) of corn-leafhopper *Dalbulus maidis* on maize plants at different time points after microbiological insecticide pulverization (*Cordyceps javanica*, strain ESALQ 1296). The columns and bars represent the mean and the standard error for each variable and time point. Bars with an asterisk (*) indicate a statistically significant difference (P < 0.05) according to t-Student test or Mann–Whitney *U* test between untreated vs. treated insects in the same time point (*ns* non-significant).

**Figure 5:**
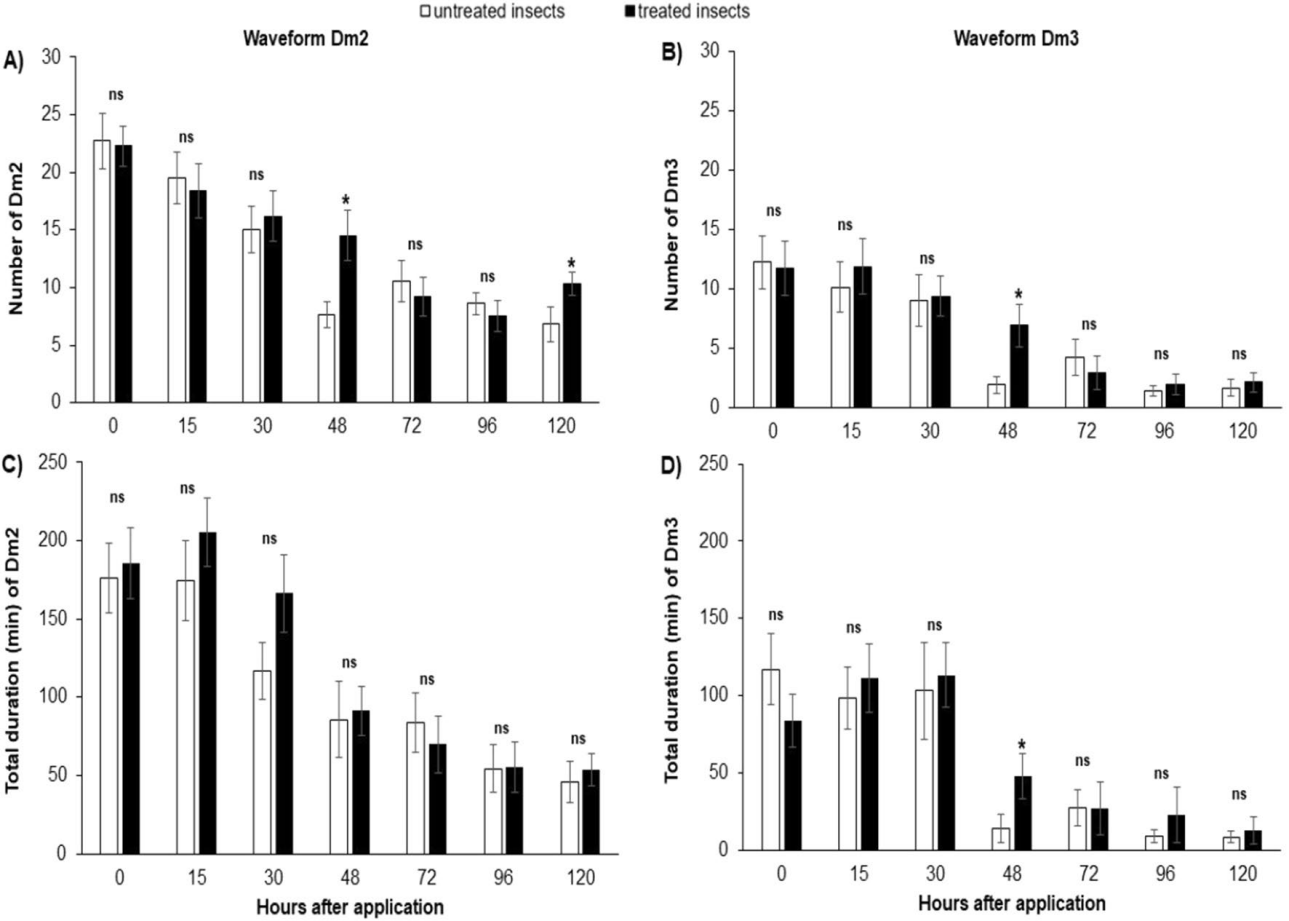
Mean number of waveform event per insect (NWEI) and total waveform duration per insect (WDI) of waveform Dm2 (**A**,**C**) and waveform Dm3 (**C**,**D**) of corn-leafhopper *Dalbulus maidis* on maize plants at different time points after microbiological insecticide pulverization (*Cordyceps javanica*, strain ESALQ 1296). The columns and bars represent the mean and the standard error for each variable and time point. Bars with an asterisk (*) indicate a statistically significant difference (P < 0.05) according to t-Student test or Mann–Whitney *U* test between untreated vs. treated insects in the same time point (*ns* non-significant).

**Figure 6:**
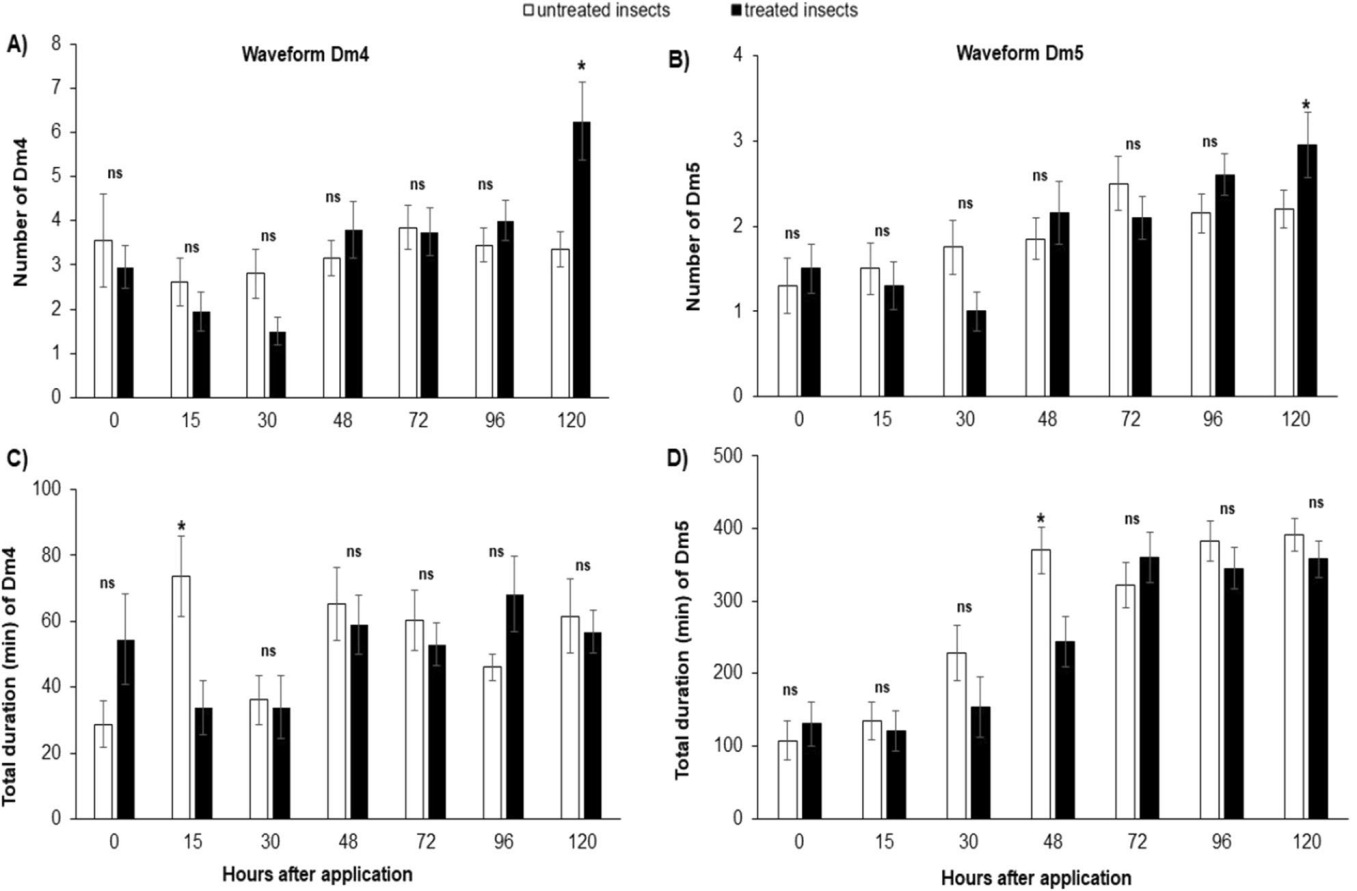
Mean number of waveform event per insect (NWEI) and total waveform duration per insect (WDI) of waveform Dm4 (**A**,**C**) and waveform Dm5 (phloem ingestion) (**C**,**D**) of corn-leafhopper *Dalbulus maidis* on maize plants at different time points after microbiological insecticide pulverization (*Cordyceps javanica*, strain ESALQ 1296). The columns and bars represent the mean and the standard error for each variable and time point. Bars with an asterisk (*) indicate a statistically significant difference (P < 0.05) according to *t* Student test or Mann–Whitney *U* test between untreated vs. treated insects in the same time point (*ns*: non-significant).

In the experiment with *D. maidis* in which insects were sprayed with fungus and immediately connected to the EPG system to record the activities, no differences were observed in non-sequential and sequential variables (Figs. 4, 5 and 6; Supplementary material Table S1).

#### 15 h after spraying

Fifteen hours after *D. maidis* were sprayed, the only observed effect was on the (total) waveform duration per insect (WDI) of the Dm4 waveform, associated with the conditioning of the insect in the phloem, where salivation and egestion occur, and with the inoculation of *S. kunkelii*^14^. Insects sprayed with the biological product based on *C. javanica* spent less duration per insect (approximately half the time) (33.8 ± 8.1 min) performing Dm4 than the leafhoppers in the control treatment (73.8 ± 21.2 min) (U = 122,000; P = 0.03) (Fig. 6). The other sequential and non-sequential parameters were not affected by application of the microbiological product (Figs. 4, 5 and 6; Supplementary material Table S2).

#### 30 h after spraying

No differences were observed in non-sequential and sequential variables at time point 30 h after spraying (P > 0.05) (Figs. 4, 5 and 6; Supplementary material Table S3).

#### 48 h after spraying

The effects of *C. javanica* on the stylet activities of *D. maidis* were more apparent when EPG recordings were started 48 h after an insect was sprayed, when practically all variables showed significant differences.

Regarding non-phloem variables, insects sprayed with the microbiological insecticide performed more Probing (NWEI) (t = – 2.33; P = 0.02), more non-probing events (waveform np) (t = − 2.44; P = 0.02), waveform Dm1 (t = − 3.0; P < 0.01) (Fig. 4), waveform Dm2 (t = − 3.3; P < 0.01), and waveform Dm3 (U = 97,000; P < 0.01) (Fig. 5) than insects in the control treatment. No effects were observed on the numbers of events for waveforms Dm4 and Dm5, which are associated with the phloem phase (Fig. 6; Supplementary material Table S4).

Insects treated with the microbiological product spent less time performing probes (U = 68,000; P < 0.01) than the control insects. However, treated insects performed longer mean durations of waveform Dm1 (U = 100,000; P < 0.01) and spent more time with the stylets outside the plant tissue (waveform np) (t = − 4.11; P < 0.01) than the untreated insects (Fig. 4). Furthermore, they spent almost four times longer performing waveform Dm3 than the insects in the control treatment (U = 99,500; P < 0.01) (Fig. 5; Supplementary material Table S4).

The phloem variables were also affected, since insects treated with the microbiological product spent shorter duration (WDI) ingesting phloem sieve (waveform Dm5) than untreated insects (t = 2.68; P = 0.01), reducing the time that treated insects spent performing all activities in the phloem (D4 + 5 = Dm4 + Dm5) (U = 96,000; P < 0.01) (Fig. 6; Supplementary material Table S4).

Regarding the sequential variables, insects treated with the microbiological product performed a larger “Number of probes for the 1st Dm4” than untreated insects, that is, treated insects performed more probes until they made the first contact with the phloem tissue than untreated ones (t = − 3.02; P < 0.01). The same trend was observed for the “Time from the beginning of the EPG record to the 1st phloem”, that is, treated insects took longer to make the first contact with the phloem from the beginning of the EPG record than the untreated insects (t = − 2.46; P 0.02), as well as for the “Time from the 1st probe to the 1st contact with the phloem” (t = − 2.24; P 0.03).

#### 72 h after spraying

In contrast to the observation 48 h after the product application, where several variables of the probing behavior of *D. maidis* were altered, 72 h after application the probing behavior of insects showed no effects (Figs. 4, 5, 6; Supplementary material Table S5).

#### 96 h after spraying

In the experiment with *D. maidis* evaluated 96 h after the application of the microbiological product, no significant differences were observed in any of the non-sequential variables analyzed, at both the phloem (Fig. 6) and non-phloem levels (P > 0.05) (Figs. 4 and 5; Supplementary material Table S6). A significant difference was observed only in one sequential variable, “Number of probes performed for the 1st Dm4”, which was larger in the untreated insects (11.7 ± 3.2) than in the treated ones (4.5 ± 1.0) (U = 100,000; P < 0.01).

#### 120 h after spraying

Five days after the microbiological application (120 h), treated insects performed the waveforms Dm2 (U = 91,500; P = 0.03) (Fig. 5), Dm4 (t = – 2.38; P = 0.02), and D5 (t = – 2.22; P = 0.03) (associated with phloem tissues and pathogen inoculation) (Fig. 6) more often (NWEI) than untreated insects. Furthermore, treated insects spent more time (WDI) performing Dm1 than untreated insects (t = – 2.56; P = 0.01) (Fig. 4; Supplementary material Table S7).

No effects were observed in the duration of activities associated with the phloem, nor in any sequential parameter.

## Discussion

Leafhoppers and other sap-sucking insects consume food by inserting their stylets into plant tissues, which makes observation of this activity with the naked eye impractical. With the EPG technique, the sucking behavior in plant tissues can be observed in detail in real time and correlated with biological activities.

The stylet probing behaviors of *D. maidis* on maize plants was monitored using a direct current (DC) EPG system. The waveforms obtained were analyze using the characterization and correlation information from previous studies on *D. maidis* and species from the same subfamily^14,15,18,20–22^, although most of these studies used the alternating current system (AC or AC-DC); or correlated with these studies, as carried out by Carpane & Catalano (who used DC)^15^. As previously observed by Wayadande & Nault^19^, leafhoppers that produce a salivary sheath also produce a very similar waveform appearance within species regardless of EPG system used. Thus, even by recording with a DC system, it was possible to recognize the waveforms obtained with those available in published studies in our output traces. Nonetheless, to thoroughly document the appearance of our DC waveforms for *D. maidis,* we provided waveform images and descriptions, herein, to complement those of Carpane & Catalano^15^.

We observed five different waveform patterns, which were identified as those found by Carpane & Catalano^15^: waveforms Dm1, Dm2, and Dm3, related to behavior in non-phloem plant tissues; and waveforms Dm4 and Dm5 (Two subphases: Dm5a and Dm5b), associated with stylet activities in phloem tissues. We used the same nomenclature as Carpane & Catalano^15^ since it is the same species, reinforcing the use of this nomenclature to encourage its use in future studies.

The waveform termed Dm1 has been referred to as “stylet pathway” or “waveform 1” by some authors^14,19^. This waveform possibly involves salivation, but also the pathway of stylets through cells of more superficial tissues, such as mesophyll, epidermis, and parenchyma^15^. Waveform Dm2 (“Dm2”^15^ or “waveform 2”^14^) has a very similar shape to “waveform G”, where the ingestion of xylem sap occurs in whiteflies, aphids, and psyllids^10,11,26–28^ and is referred to as “non-sieve element ingestion”^19^. Ingestion of non-phloem cell content, which may be xylem or mesophyll, possibly occurs^21^ since honeydew secretion was observed while the insects were performing this behavior.

Waveform Dm3 has a different pattern from those previously observed for other hemipterans. It is not known exactly with which biological behavior it is associated, and we agree with other investigators that it can be considered “non-vascular behavior”. However, it usually occurs linked to waveform Dm2 (before or after)^14,19^.

Regarding behavior in the phloem tissue, waveform Dm4 refers to the first contact with phloem cells and is referred to as “X wave” (or “waveform X") by some authors^14,27^ or “Dm4”^15^. It is associated with the “phloem conditioning” phase where both salivation and passive sap ingestion would occur, since spiroplasma transmission was observed during this waveform^14^. Backus et al.^29^ stated that during the “X wave” leafhoppers perform salivation, ingestion, and egestion of functional foregut contents. Changes in this behavior by insecticides can reduce or prevent the transmission of phytopathogens such as *S. kunkelii.*

On the other hand, waveform Dm5 refers to “ingestion of phloem sap”, and as mentioned for waveform Dm2, honeydew secretion was also observed during this behavior^18,19,30^.

Wayadande & Nault^19^ reported that relatively few *D. maidis* leafhoppers ingested phloem sap (11 of 19, approximately 58%), while in the present study, of the 22 insects analyzed, 14 performed waveform Dm5 (63.6%), associated with ingestion of phloem sieve. As noted by the previous authors, this species may need a longer period to start this activity, because they evaluated the insects for only 3 h (180 min). Our data support this interpretation because our insects, on average, took 313.97 min (± 5 h) to make the first contact with phloem cells after the EPG recording began (Table 1). However, Carpane et al.^14^ observed that in their study, about 80% of the insects were able to ingest phloem sieve.

Our comparison of waveforms produced by *D. maidis* using the DC-EPG system indicates that these leafhoppers ingestion not only phloem sap but also on the content of other tissues, as evidenced by the secretion of honeydew when performing waveform Dm2, although they spend long periods ingesting phloem sap (Dm5). Our study, the most detailed ever carried out with *D. maidis* in the DC-EPG system, opens the possibility for additional studies with this species. Despite the great progress in studies of stylet probing behavior with *D. maidis*, further research is needed to better understand some behaviors.

After our comparison of waveform appearance, we used this method to determine the effects of the entomopathogenic fungus *C. javanica* on the stylet probing and ingestion behavior of *D. maidis*. We sought to know in detail whether this biological control agent is capable of changing leafhopper probing variables, and how this occurs, to evaluate whether this control strategy could be useful as a tool in integrated management of this pest.

Several studies have reported that fungi of the order Hypocreales, such as *C. javanica,* act as important control agents for several groups of sap-sucking insects of the order Hemiptera^6,9,31–33^. The behavior and performance of these insects after contact with the fungus is still little understood, especially regarding insect vectors of phytopathogens, which ingest from on phloem sieve elements. These studies mainly aimed to evaluate insect mortality, and few have studied the effects on probing behavior using the EPG technique^9,33,34^.

In general, entomopathogenic microorganisms require a certain time period to effectively infect and kill their host, and during this period these insects continue to cause damage, both direct and indirect, transmitting pathogens to various plants^35^. Because of the time required by these control agents, many producers’ resorts to chemical control, which, although it can cause several problems, can reduce a pest population quickly. However, many insecticides are not capable of altering the stylet activities of their hosts, and despite causing high mortality, they do not reduce the spread of diseases in the field.

Maluta et al.^9^ observed that the stylet activities of the citrus psyllid *Diaphorina citri* Kuwayama were considerably altered 30 to 96 h after application of *C. fumosorosea.* The authors found that insects treated with a product based on the entomopathogenic fungus showed significant alterations in the parameters associated with the phloem, which could be an important tool in integrated management of pests (IPM) and diseases.

When evaluating the stylet probing behavior of the aphid *Aphis gossypii* Glover (Hemiptera: Aphididae), Gonzáles-Mas et al.^33^ concluded that endophytic colonization by the fungus *B. bassiana* can be used in IPM to reduce the transmission of some viruses by *A. gossypii*, since they observed a reduction in the transmission of Cucurbit aphid-borne yellow virus (CABYV) (Polerovirus) and Cucumber mosaic virus (CMV) (Cucumovirus) in melon plants.

Carpane et al.^14^ observed that the acquisition and inoculation of *Spiroplasma kunkelii* occurs during the phloem phase, both in the initial conditioning phase (DM4) and in sap ingestion (Dm5). Thus, alterations in the activities associated with the phloem can substantially reduce the transmission of this phytopathogen, as well as reduce the transmission of other pathogens restricted to the phloem transmitted by *D. maidis*^1,3^.

We observed that considerable changes in the probing behavior of the maize leafhopper occurred mainly 48 h after the application, in non-phloem parameters, as evidenced by the larger number of times that these insects performed D2 and D3 and by the longer time spent performing D3, as well as in relation to the ingestion of phloem sap, since the insects spent much less time ingesting sap from these vessels. Few differences were observed at the time points before and after 48 h. Although in the first hours after the microbiological application, the fungus is still showing no perceptible effect, after 48 h there was a significant reduction in the ingestion of phloem sieve, which could result in less acquisition of pathogens limited to these vessels.

Bacteria transmitted by *D. maidis* colonize the phloem vessels, being acquired, and inoculated in this tissue as well, which requires a long period of probing activity occur the acquisition and inoculation (hours to days). Furthermore, the latency period of these pathogens is significantly long (weeks to months), and only after this period are insects capable of transmitting the phytoplasma^36,37^.

Although no significant differences were observed after 48 h, for our studies, we used only insects that were alive at the time of the experiment but were proven to be infected with the fungus. On the other hand, in the field, the insects would probably be affected much sooner due to the microclimate in the maize crops, causing the insects to become infected and die.

According to Carpane & Catalano^15^, waveform Dm3 refers to a mechanical activity of the stylets, where the insects would be moving but keeping the stylets inserted in the plant tissue. The more numerous periods of Dm3 and the longer time spent in this activity in the treated insects may indicate a certain resistance or mechanical difficulty of the insects in probing, since we also observed that 48 h after the application of the fungus, these insects remained three times longer with their stylets outside the plant tissue than untreated insects and spent less time ingesting the phloem contents.

Our study revealed details of the *D. maidis* waveform in the DC-EPG system in maize plants, demonstrating that leafhoppers of the subfamily Deltocephalinae show similar behavior using all EPG system, that is, AC, DC and AC-DC. Furthermore, our studies have shown that the active ingredient in *C. javanica* present in the commercial microbiological product affects the probing behavior of *D. maidis*, mainly 48 h after its application, which may reduce the probing activities of this insect in maize plants, and consequently reduce the transmission of pathogens by this species.

### Supplementary Information

Supplementary Tables.

## Data Availability

Data sets used or analyzed during current study are available from the corresponding author on reasonable request.
