# Supplementary material for: DC-electrical penetration graph waveforms for Dalbulus maidis (Hemiptera: Cicadellidae) and the effects of entomopathogenic fungi on its probing behavior
Source: Sci Rep. 2023 Dec 12;13:22033. doi: 10.1038/s41598-023-48779-x (PMC10716247; doi:10.1038/s41598-023-48779-x)
Supplement: Supplementary file 1 — Supplementary Tables. [file 41598_2023_48779_MOESM1_ESM.pdf]

DC-electrical penetration graph waveforms for *Dalbulus maidis* (Hemiptera: Cicadellidae) and the effects of entomopathogenic fungi on its probing behavior

Nathalie Maluta[1,2] *, Thiago Castro[2], João Roberto Spotti Lopes[1]

[1]Department of Entomology and Acarology, Luiz de Queiroz College of Agriculture, University of São Paulo, C.P. 9, 13418-900, Piracicaba, SP, Brazil; nathaliepradomaluta@gmail.com; jrslopes@usp.br
[2] Koppert Biological Systems, Rodovia Margarida da Graça Martins s/n - Km 17,5, 13400-970, Piracicaba, SP, Brazil tcastro@koppert.com.br

*****Correspondence: nathaliepradomaluta@gmail.com ; Tel: +55 19 3429-4199 r.214 (N.M)

Table S1. Mean (± SEM) of non-sequential and sequential EPG variables for 10-h recordings of the probing behavior of *Dalbulus maidis* on maize (*Zea mays*) plants immediately after (time point 0h) sprayed with Octane® *(Cordyceps javanica).*

**EPG non-sequential variables**	**Control 0h n= 20**	**Octane 0h n= 20**	***P*-value[c]**	**U or t value**
**NWEI[a]**				
Probe	23.10 ± 4.26	25.85 ± 2.40	0.148	-1.475
np	23.30 ± 4.24	26.15 ± 2.40	0.145	-1.488
Dm1	40.95 ± 6.31	44.95 ± 3.69	0.587	-0.547
Dm2	22.75 ± 2.41	22.30 ± 1.73	0.880	0.151
Dm3	12.25 ± 223	11.75 ± 2.23	0.843	0.199
Dm4	3.55 ± 1.06	2.95 ± 0.49	0.774	189.500
Dm5	1.30 ± 0.32	1.50 ± 0.29	0.519	177.000
Dm5s (Dm5 > 10 min)	0.85 ± 0.22	1.25 ± 0.25	0.204	155.500
**WDI (minutes)[b]**				
Probe	520.17 ± 8.47	528.88 ± 20.50	0.160	148.000
np	79.83 ± 8.47	82.79 ± 15.90	0.194	152.000
Dm1	90.51 ± 11.76	81.50 ± 7.68	0.956	0.056
Dm2	176.34 ± 22.15	185.49 ± 22.64	0.774	-0.289
Dm3	117.07 ± 23.06	84.07 ± 17.15	0.121	142.500
Dm4	28,86 ± 7.04	54.53 ± 13.68	0.266	369.000
Dm5	107.39 ± 26.94	131.05 ± 30.51	0.668	184.500
Total duration of phloem phase	136.25 ± 30.14	185.58 ± 37.00	0.481	174.000
**Sequential variables**				
Time to 1st probe from start of EPG (min)	4.61 ± 1.31	3.27 ± 0.51	0.891	-0.137
Number of probes to the 1st phloem	10.30 ± 1.83	12.35 ± 1.61	0.249	-1.170
Time from start of EPG to 1st phloem (min)	315.46 ± 48.37	282.33 ± 48.59	0.664	184.000
Number of probes after 1st phloem	12.80 ± 4.32	13.50 ± 2.35	0.253	158.500
Time from 1st probe to 1st phloem (min)	310.85 ± 48.40	279.07 ± 48.43	0.725	187.000

[a] **NWEI,** number of waveform events per insect; [b] **WDI,** total waveform duration (min) per insect. [c] Statistical comparisons between treatments for each parameter were made by Student´s t-test for Gaussian distribution variables or non-parametric Mann-Whitney U-test for non-Gaussian distribution variables. Underline-type indicates significant differences ($P < 0.05$) (Backus et al. 2007).

Table S2. Mean (± SEM) of non-sequential and sequential EPG variables for 10-h recordings of the probing behavior of *Dalbulus maidis* on maize (*Zea mays*) plants 15 hours after sprayed with Octane® *(Cordyceps javanica).*

**EPG non-sequential variables**	**Control 15h n= 20**	**Octane 15h n= 20**	***P*-value[c]**	**U or t value**
**NWEI[a]**				
Probe	21.10 ± 3.02	17.95 ± 3.13	0.333	0.980
np	21.10 ± 3.02	18.30 ± 3.13	0.445	171.000
Dm1	35.20 ± 4.08	29.75 ± 4.17	0.360	0.926
Dm2	19.50 ± 2.25	18.40 ± 2.31	0.647	0.462
Dm3	10.40 ± 2.12	11.90 ± 2.37	0.383	-0.882
Dm4	2.60 ± 0.54	1.95 ± 0.44	0.327	163.500
Dm5	1.50 ± 0.30	1.30 ± 0.28	0.678	184.000
Dm5s (Dm5 > 10 min)	1.30 ± 0.27	1.15 ± 0.27	0.659	183.000
**WDI (minutes)[b]**				
Probe	555.10 ± 11.75	523.98 ± 15.69	0.068	132.000
np	44.90 ± 11.75	76.01 ± 15.69	0.141	-1.503
Dm1	73.63 ± 13.91	53.19 ± 6.0	0.253	157.000
Dm2	174.57 ± 25.77	205.52 ± 22.09	0.409	-0.835
Dm3	98.50 ± 20.16	111.50 ± 22.09	0.602	180.000
Dm4	73.77 ± 21.16	33.85 ± 8.13	0.035	122.000
Dm5	134.64 ± 25.89	120.37 ± 27.36	0.620	181.000
Total duration of phloem phase	208.41 ± 33.08	154.22 ± 33.50	0.242	156.000
**Sequential variable**				
Time to 1st probe from start of EPG (min)	1.35 ± 0.20	6.09 ± 3.06	0.072	133.000
Number of probes to the 1st phloem	9.30 ± 1.37	10.70 ± 2.84	0.738	0.337
Time from start of EPG to 1st phloem (min)	267.45 ± 47.23	331.20 ± 55.07	0.341	164.000
Number of probes after 1st phloem	11.9 ± 3.50	7.25 ± 2.14	0.529	176.000
Time from 1st probe to 1st phloem (min)	266.10 ± 47.23	325.11 ± 55.24	0.414	167.000

[a] **NWEI,** number of waveform events per insect; [b] **WDI,** total waveform duration (min) per insect. [c] Statistical comparisons between treatments for each parameter were made by Student´s t-test for Gaussian distribution variables or non-parametric Mann-Whitney U-test for non-Gaussian distribution variables. Underline-type indicates significant differences ($P < 0.05$) (Backus et al. 2007).

Table S3. Mean (± SEM) of non-sequential and sequential EPG variables for 10-h recordings of the probing behavior of *Dalbulus maidis* on maize (*Zea mays*) plants 30 hours after sprayed with Octane® *(Cordyceps javanica).*

**EPG non-sequential variables**	**Control 30h n= 20**	**Octane 30h n= 20**	***P*-value**[c]	**U or t value**
**NWEI**[a]				
Probe	13.45 ± 1.18	15.60 ± 2.29	0.995	0.006*
np	13.50 ± 1.18	15.95 ± 2.31	0.408	169.500
Dm1	25.20 ± 1.98	27.05 ± 3.39	0.640	0.471
Dm2	15.05 ± 2.01	16.20 ± 2.18	0.700	-0.388
Dm3	9.05 ± 2.17	9.40 ± 1.68	0.642	-0.469
Dm4	2.80 ± 0.56	1.50 ± 0.32	0.077	136.000
Dm5	1.75 ± 0.32	1.0 ± 0.23	0.098	141.000
Dm5s (Dm5 > 10 min)	1.60 ± 0.32	0.95 ± 0.23	0.144	148.000
**WDI**[b]				
Probes	541.79 ± 13.73	522.84 ± 16.72	0.265	158.000
np	58.21 ± 13.73	77.16 ± 16.72	0.537	-0.622
Dm1	57.29 ± 10.56	55.54 ± 11.63	0.665	184.000
Dm2	116.67 ± 18.25	166.47 ± 24.87	0.168	149.000
Dm3	103.26 ± 31.38	113.34 ± 21.13	0.791	-0.266
Dm4	36.20 ± 7.49	33.94 ± 9.49	0.444	17.000
Dm5	228.27 ± 38.12	153.56 ± 42.05	0.132	145.000
Total duration of phloem phase	264.47 ± 42.98	187.49 ± 46.07	0.172	150.000
**Sequential variables**				
Time to 1st probe from start of EPG (min)	1.38 ± 0.15	2.73 ± 1.37	0.871	194.000
Number of probes to the 1st phloem	8.40 ± 1.07	8.85 ± 1.29	0.790	-0.268
Time from start of EPG to 1st phloem (min)	248.78 ± 50.84	311.18 ± 56.41	0.566	179.000
Number of probes after 1st phloem	5.05 ± 1.36	6.75 ± 1.98	0.933	197.000
Time from 1st probe to 1st phloem (min)	247.40 ± 50.823	308.44 ± 56.63	0.589	180.000

[a] **NWEI,** number of waveform events per insect; [b] **WDI,** total waveform duration (min) per insect. [c] Statistical comparisons between treatments for each parameter were made by Student´s t-test for Gaussian distribution variables or non-parametric Mann-Whitney U-test for non-Gaussian distribution variables. Underline-type indicates significant differences ($P < 0.05$) (Backus et al. 2007).

Table S4. Mean (± SEM) of non-sequential and sequential EPG variables for 10-h recordings of the probing behavior of *Dalbulus maidis* on maize (*Zea mays*) plants 48 hours after sprayed with Octane® *(Cordyceps javanica).*

EPG non-sequential variables	Control 48h n= 20	Octane 48h n= 20	*P*-value[c]	U or t value
**NWEI[a]**				
Probe	13.0 ± 1.78	18.90 ± 1.79	0.025	-2.333
np	13.05 ± 1.80	19.20 ± 1.76	0.019	-2.443
Dm1	21.45 ± 2.62	32.80 ± 2.72	0.005	-3.006
Dm2	7.65 ± 1.14	14.50 ± 2.19	0.007	-3.341
Dm3	1.95 ± 0.73	6.95 ± 1.79	0.004	97.000
Dm4	3.15 ± 0.39	3.80 ± 0.64	0.436	171.500
Dm5	1.85 ± 0.24	2.15 ± 0.37	0.615	182.000
Dm5s (Dm5 > 10 min)	1.70 ± 0.19	2.15 ± 0.37	0.416	171.000
**WDI[b]**				
Probes	572.02 ± 8.40	502.42 ± 23.97	< 0.01	68.000
np	27.98 ± 8.40	97.58 ± 23.97	< 0.01	-4.107
Dm1	37.76 ± 4.82	76.11 ± 16.81	0.007	100.000
Dm2	85.46 ± 24.33	91.52 ± 15.62	0.072	-1.852
Dm3	13.82 ± 9.01	48.01 ± 14.46	0.005	99.500
Dm4	65.24 ± 10.93	58.97 ± 8.84	0.850	193.000
Dm5	369.74 ± 31.68	244.31 ± 34.48	0.011	2.679
Total duration of phloem phase	434.98 ± 30.96	303.28 ± 40.64	0.005	96.000
**Sequential variables**				
Time to 1st probe from start of EPG (min)	1.66 ± 0.24	8.73 ± 3.63	0.224	155.000
Number of probes to the 1st phloem	4.5 ± 0.77	9.40 ± 1.55	0.005	-3.016
Time from start of EPG to 1st phloem (min)	87.11 ± 25.79	200.82 ± 51.22	0.019	-2.460
Number of probes after 1st phloem	8.50 ± 1.68	9.50 ± 1.62	0.744	188.000
Time from 1st probe to 1st phloem (min)	85.45 ± 25.85	192.09 ± 50.62	0.031	-2.242

[a] **NWEI,** number of waveform events per insect; [b] **WDI,** total waveform duration (min) per insect. [c] Statistical comparisons between treatments for each parameter were made by Student´s t-test for Gaussian distribution variables or non-parametric Mann-Whitney U-test for non-Gaussian distribution variables. Underline-type indicates significant differences (*P* < 0.05) (Backus et al. 2007).

Table S5. Mean (± SEM) of non-sequential and sequential EPG variables for 10-h recordings of the probing behavior of *Dalbulus maidis* on maize (*Zea mays*) plants 72 hours after sprayed with Octane® *(Cordyceps javanica).*

EPG non-sequential variables	Control 72h n= 20	Octane 72h n= 20	*P*-value[c]	U or t value
**NWEI[a]**				
Probes	16.55 ± 2.40	20.40 ± 3.86	0.823	0.226
np	16.75 ± 2.41	20.45 ± 3.86	0.787	0.273
Dm1	27.10 ± 3.06	31.15 ± 5.02	0.495	0.688
Dm2	10.55 ± 1.79	9.20 ± 1.69	0.395	0.861
Dm3	4.25 ± 1.53	2.95 ± 1.37	0.130	146.500
Dm4	3.85 ± 0.51	3.75 ± 0.55	0.795	0.261
Dm5	2.50 ± 0.32	2.10 ± 0.25	0.386	169.000
Dm5s (Dm5 > 10 min)	2.30 ± 0.30	2.05 ± 0.23	0.652	184.000
**WDI[b]**				
Probes	550.69 ± 12.95	559.60 ± 12.85	0.372	167.000
np	49.31 ± 12.95	40.40 ± 12.85	0.251	1.167
Dm1	57.37 ± 8.26	49.10 ± 5.16	0.766	189.000
Dm2	83.83 ± 19.16	69.77 ± 17.84	0.401	0.849
Dm3	27.53 ± 11.54	26.74 ± 17.16	0.081	138.000
Dm4	60.39 ± 9.12	53.01 ± 6.53	0.515	0.658
Dm5	321.57 ± 31.51	360.98 ± 34.76	0.406	-0.840
Total duration of phloem phase	381.96 ± 34.55	413.99 ± 34.91	0.516	176.000
**Sequential variables**				
Time to 1st probe from start of EPG (min)	1.94 ± 34.55	1.79 ± 0.30	0.465	0.738
Number of probes to the 1st phloem	4.40 ± 0.69	6.45 ± 1.49	0.744	188.000
Time from start of EPG to 1st phloem (min)	73.16 ± 17.90	85.05 ± 29.81	0.927	-0.092
Number of probes after 1st phloem	12.15 ± 2.25	13.95 ± 2.99	0.850	193.000
Time from 1st probe to 1st phloem (min)	71.22 ± 17.88	83.26 ± 29.87	0.969	-0.039

[a] **NWEI,** number of waveform events per insect; [b] **WDI,** total waveform duration (min) per insect. [c] Statistical comparisons between treatments for each parameter were made by Student´s t-test for Gaussian distribution variables or non-parametric Mann-Whitney U-test for non-Gaussian distribution variables. Underline-type indicates significant differences ($P < 0.05$) (Backus et al. 2007).

Table S6. Mean (± SEM) of non-sequential and sequential EPG variables for 10-h recordings of the probing behavior of *Dalbulus maidis* on maize (*Zea mays*) plants 96 hours after sprayed with Octane® *(Cordyceps javanica).*

EPG non-sequential variables	Control 96h n= 20	Octane 96h n= 20	*P*-value[c]	U or t value
**NWEI[a]**				
Probes	25.35 ± 4.67	17.75 ± 2.40	0.148	1.477
np	25.50 ± 4.67	18.05 ± 2.39	0.169	1.401
Dm1	36.40 ± 5.25	27.95 ± 3.24	0.160	1.432
Dm2	8.60 ± 0.99	7.55 ± 1.32	0.248	1.173
Dm3	1.45 ± 0.41	2.0 ± 0.90	0.567	180.500
Dm4	3.45 ± 0.39	4.0 ± 0.45	0.365	-0.916
Dm5	2.15 ± 0.23	2.60 ± 0.24	0.139	148.000
Dm5s (Dm5 > 10 min)	2.05 ± 0.22	2.45 ± 0.25	0.239	158.500
**WDI[b]**				
Probes	554.38 ± 10.84	542.50 ± 16.91	0.725	187.000
np	45.62 ± 10.84	54.53 ± 14.87	0.656	-0.449
Dm1	62.19 ± 8.71	50.88 ± 5.60	0.455	0.754
Dm2	54.32 ± 15.41	55.42 ± 15.87	0.382	0.885
Dm3	9.03 ± 3.90	22.50 ± 17.77	0.483	176.000
Dm4	46.19 ± 4.03	68.33 ± 11.36	0.140	145.500
Dm5	382.64 ± 27.46	345.37 ± 28.60	0.273	159.500
Total duration of phloem phase	428.83 ± 29.40	413.70 ± 30.90	0.655	183.500
**Sequential variables**				
Time to 1st probe from start of EPG (min)	3.18 ± 1.68	4.33 ± 1.08	0.05	125.000
Number of probes to the 1st phloem	11.70 ± 3.20	4.50 ± 1.02	<u>0.006</u>	100.000
Time from start of EPG to 1st phloem (min)	86.49 ± 29.53	80.05 ± 32.93	0.116	1.609
Number of probes after 1st phloem	13.65 ± 3.40	13.25 ± 2.24	0.560	178.500
Time from 1st probe to 1st phloem (min)	83.31 ± 28.18	75.71 ± 32.42	0.065	1.903

[a] **NWEI,** number of waveform events per insect; [b] **WDI,** total waveform duration (min) per insect. [c] Statistical comparisons between treatments for each parameter were made by Student´s t-test for Gaussian distribution variables or non-parametric Mann-Whitney U-test for non-Gaussian distribution variables. Underline-type indicates significant differences (*P* < 0.05) (Backus et al. 2007).

Table S7. Mean (± SEM) of non-sequential and sequential EPG variables for 10-h recordings of the probing behavior of *Dalbulus maidis* on maize (*Zea mays*) plants 120 hours after sprayed with Octane® *(Cordyceps javanica).*

EPG non-sequential variables	Control 120h n= 20	Octane 120h n= 20	*P*-value[c]	U or t value
**WDE[a]**				
Probes	18.0 ± 2.99	18.10 ± 1.87	0.379	167.500
np	18.10 ± 2.98	18.15 ± 1.87	0.989	-0.014
Dm1	26.65 ± 4.12	33.0 ± 2.52	0.196	-1.317
Dm2	6.85 ± 1.51	10.30 ± 0.99	0.003	91.500
Dm3	1.70 ± 0.71	2.15 ± 0.85	0.320	165.000
Dm4	3.35 ± 0.39	6.25 ± 0.88	0.023	-2.372
Dm5	2.20 ± 0.22	3.6 ± 0.47	0.032	-2.226
Dm5s (Dm5 > 10 min)	2.10 ± 0.22	2.95 ± 0.38	0.058	132.000
**WDI[b]**				
Probes	556.23 ± 5.69	551.69 ± 11.14	0.589	180.000
np	43.77 ± 5.69	48.31 ± 11.14	0.675	0.422
Dm1	49.66 ± 4.60	70.71 ± 6.73	0.014	-2.583
Dm2	45.64 ± 13.26	53.78 ± 10.38	0.316	-1.016
Dm3	8.32 ± 3.96	12.62 ± 8.79	0.499	176.000
Dm4	61.57 ± 11.30	56.84 ± 6.41	0.725	187.000
Dm5	391.03 ± 22.98	357.74 ± 24.63	0.137	145.000
Total duration of phloem phase	452.60 ± 19.57	414.58 ± 26.28	0.079	135.000
**Sequential variables**				
Time to 1st probe from start of EPG (min)	10.44 ± 3.63	1.68 ± 0.23	0.871	194.000
Number of probes to the 1st phloem	7.50 ± 1.10	4.75 ± 0.85	0.086	1.763
Time from start of EPG to 1st phloem (min)	59.37 ± 8.03	68.30 ± 29.47	0.266	1.130
Number of probes after 1st phloem	10.50 ± 2.38	13.35 ± 1.80	0.159	148.000
Time from 1st probe to 1st phloem (min)	48.93 ± 7.04	66.62 ± 29.48	0.487	0.702

[a] **NWEI,** number of waveform events per insect; [b] **WDI,** total waveform duration (min) per insect. [c] Statistical comparisons between treatments for each parameter were made by Student´s t-test for Gaussian distribution variables or non-parametric Mann-Whitney U-test for non-Gaussian distribution variables. Underline-type indicates significant differences ($P < 0.05$) (Backus et al. 2007).
